# Influence of Different Light Regimes on the Mycoparasitic Activity and 6-Pentyl-α-pyrone Biosynthesis in Two Strains of *Trichoderma atroviride*

**DOI:** 10.3390/pathogens9100860

**Published:** 2020-10-21

**Authors:** Dubraska Moreno-Ruiz, Alessandro Fuchs, Kristina Missbach, Rainer Schuhmacher, Susanne Zeilinger

**Affiliations:** 1 Department of Microbiology, University of Innsbruck, 6020 Innsbruck, Austria; Dubraska.Moreno-Ruiz@uibk.ac.at (D.M.-R.); a.fuchs2323@gmail.com (A.F.); 2 Department of Agrobiotechnology (IFA-Tulln), University of Natural Resources and Life Sciences Vienna (BOKU), 1180 Tulln, Austria; Kristina.missbach@boku.ac.at (K.M.); rainer.schuhmacher@boku.ac.at (R.S.)

**Keywords:** *Trichoderma atroviride*, mycoparasitism, secondary metabolites, 6-pentyl-α-pyrone, Tmk3 MAP kinase, light, dark

## Abstract

The ascomycete *Trichoderma atroviride* is well known for its mycoparasitic lifestyle. Similar to other organisms, light is an important cue for *T. atroviride.* However, besides triggering of conidiation, little is known on the physiological responses of *T. atroviride* to light. In this study, we analyzed how cultivation under different light wavelengths and regimes impacted the behavior of two *T. atroviride* wild-type strains: IMI206040 and P1. While colony extension of both strains was slightly affected by light, massive differences in their photoconidation responses became evident. *T. atroviride* P1 colonies conidiated under all conditions tested including growth in complete darkness, while IMI206040 required white, blue or green light to trigger asexual reproduction. Interestingly, deletion of the stress-activated MAP kinase-encoding gene *tmk3* abolished the ability of strain P1 to conidiate in red and yellow light as well as in darkness. Furthermore, light-dependent differences in the mycoparasitic activity and in the biosynthesis of the secondary metabolite 6-pentyl-α-pyrone (6-PP) became evident. 6-PP production was highest upon dark incubation, while light, especially exposure to white light as light/dark cycles, had an inhibitory effect on its biosynthesis. We conclude that the response of *T. atroviride* to light is strain-dependent and impacts differentiation, mycoparasitism, and 6-PP production; hence, this should be considered in experiments testing the mycoparasitic activity of these fungi.

## 1. Introduction

The mycoparasitic fungus *Trichoderma atroviride* is applied in agriculture to protect plants against a variety of fungal pathogens. The mycoparasitic attack comprises several mechanisms such as the production of antifungal metabolites and hydrolytic enzymes and is largely affected by environmental conditions [1]. Much of our knowledge on fungal mycoparasitism comes from studies with *T. atroviride*, of which two strains are frequently used as model organisms: *T. atroviride* IMI206040, a strain isolated from a plum tree in an orchard in southern Sweden, and *T. atroviride* P1 (ATCC 74058), a fungicide resistant isolate from the UK. Both *T. atroviride* strains are known as producers of 6-pentyl-α-pyrone (6-PP), a strongly antifungal secondary metabolite, which also shows plant-growth promoting characteristics, although in a concentration-dependent manner. In addition, 6-PP is responsible for the characteristic “coconut aroma” of certain *Trichoderma* species [2,3]. However, the biosynthetic route and genes involved in the biosynthesis of 6-PP still are elusive [4]. Responses to environmental stimuli such as nutrient availability, stress, host-derived cues, and light are known to lead to a cellular adaptation process in fungi that comprises activation of signaling pathways in order to regulate cellular outcomes. Regulated processes include primary and secondary metabolism, morphology, sexual and asexual development, and virulence functions [5]. The sensing of light by filamentous fungi has mostly been explored in *Neurospora crassa* and *Aspergillus* spp., with the best characterized photosensory proteins being involved in blue light sensing [6,7,8,9,10,11]. The two main *N. crassa* blue light photoreceptors are White Collar-1 (WC-1) and White Collar-2 (WC-2), which assemble into the White Collar Complex (WCC) that, after illumination, acts as a transcriptional regulator of light-activated genes [12,13,14]. In many fungi, blue light influences conidiation, morphology, stress signaling, the production of secondary metabolites, and DNA repair. However, effects of blue light significantly vary among different fungal species. Blue light, for example, leads to the suppression of conidiation and enhanced virulence in *Botrytis cinerea* [15], while it induces conidiation in species of *Trichoderma* [16,17]. In *T. atroviride*, the stress-activated MAP kinase Tmk3 undergoes rapid phosphorylation when the fungus is exposed to light, and signaling via Tmk3 was reported to act in concert with the Blr photoreceptor complex (WCC homologue; consisting of Blr-1 and Blr-2) in the activation of expression of genes regulated by blue light [18].

The effect of red light is best studied in *Aspergillus* spp. where red light regulates the balancing between asexual and sexual development [11,19]. The single *A. nidulans* red light sensing phytochrome FphA was shown to interact with the blue light sensor complex WCC and the secondary metabolism regulator VeA, revealing that fungal physiology is affected by a crosstalk between red and blue light response pathways [20,21]. In *Trichoderma reesei* red light had no effect on conidiation and is, hence, considered as “safe light” for the manipulation of these fungi in dark experiments [22,23], while in *T. atroviride* strain IMI206040, red light could activate several genes that are related to glucanase production induced during mycoparasitism [24]. Green light is sensed by membrane-integral opsins such as *N. crassa* NOP-1 [25,26,27] and *Fusarium fujikuroi* CarO [28]. Such microbial opsins are encoded in several fungal genomes. Interestingly, however, among the three best-characterized *Trichoderma* species, an opsin-encoding gene is present only in the *T. atroviride* genomes while it is missing in *T. reesei* and *T. virens* [29,30]. Although fungal green light sensing and signaling is poorly understood, CarO seems to affect spore germination in light in *F. fujikuroi*, and NOP-1 emerged as a regulator of sexual reproduction and expression of conidiation-related genes in *N. crassa* [31,32].

Similar to most other filamentous fungi, *Trichoderma* species have three mitogen-activated protein kinases (MAPKs) designated Tmk1, Tmk2, and Tmk3. Tmk3 (Hog1) is part of the conserved stress-activated protein kinase (SAPK) signaling cascade that governs pathogenicity, asexual reproduction, and circadian rhythms in response to osmotic-, oxidative-, heavy metal-, and injury-caused stress in various fungi [6,33,34,35,36,37,38,39,40,41]. Light is another environmental cue that triggers the SAPK pathway [18,41,42]. In *T. atroviride* IMI206040, Tmk3 phosphorylation is connected to Blr-1-dependent blue light sensing [18], and in *A. nidulans*, initiation of the pathway by red light has been reported [42].

Cultivation of *Trichoderma* fungi under light–dark cycles or in complete darkness usually is used to study conidiation and mycoparasitism. *Trichoderma* species were described to infinitely grow as mycelium in complete darkness, while conidia are continuously produced across the colony in constant light. In addition, the response to light–dark cycles is characterized by concentric rings of conidia. A defined light pulse in contrast leads at the colony margin to a single conidiation ring [43,44,45], and blue light pulses were shown to promote changes in the plasma membrane potential, intracellular ATP levels, and adenylate cyclase activity [46]. A recent report describes that the *T. atroviride* strains IMI206040 and P1 display phenotypic differences when grown under light or dark conditions [47]. Besides that, only little information exists on the influence of light on *Trichoderma* mycoparasitism. Changes in the mycoparasitic behavior and secondary metabolite production between light and dark conditions were reported for *T. atroviride* strains IMI206040 and P1 [47], but there are no studies available on the response of this fungus to light of defined wavelengths.

In the present study, we describe how cultivation under different light wavelengths affects the behavior of the two *T. atroviride* strains IMI206040 and P1. We provide data on their light-dependent radial growth, conidiation behavior, and mycoparasitic activities as well as the influence of the different light regimes on the production of 6-pentyl-α-pyrone (6-PP), one of the main antifungal secondary metabolites derived from *T. atroviride*. In addition, we assessed the role of the Tmk3 MAP kinase in these processes by employing respective Δ*tmk3* deletion mutants of both strains.

## 2. Materials and Methods

### 2.1. Strains and Growth Conditions

*Trichoderma atroviride* strains IMI206040 (ATCC 20476) and P1 (ATCC 74058) as well as the plant pathogens *Botrytis cinerea* B05.10, *Fusarium oxysporum* f. sp. *lycopersici* strain 4287, and *Rhizoctonia solani* (pathogenic isolate obtained from the collection of the Institute of Plant Pathology, Università degli Studi di Napoli “Federico II”, Naples, Italy), were used in this study. Fungi were cultivated and maintained on potato dextrose agar (PDA; Becton, Dickinson and Company, Le Pont De Claix, France) at 25 °C in darkness or under different light regimes. The Δ*tmk3* mutant strain derived from IMI206040 [18] was maintained in the presence of 200 µg/mL Hygromycin B (Calbiochem^®^, Merck KGaA, Darmstadt, Germany).

### 2.2. Generation of tmk3 Gene Deletion Mutants of T. atroviride P1

To knock-out the *tmk3* gene, *T. atroviride* P1 was transformed with a deletion cassette bearing 1 kb of the 5′ and 3′ noncoding regions of *tmk3* flanking the *hph* (Hygromycin B mediating resistance) cassette obtained from plasmid pGFP-XYR1 [48]. Primers used for DNA fragment amplification and assembly with the NEBuilder^®^ Hifi DNA Assembly Kit (New England Biolabs, Ipswich, MA, USA) are given in Supplementary Table S1. Emerging transformants were selected on PDA in the presence of 200 µg/mL Hygromycin B (Calbiochem^®^, Merck KG, Darmstadt, Germany). To reach mitotic stability, transformants were purified by three rounds of single spore isolation. Deletion of *tmk3* and integration of the hygromycin resistance cassette at the target locus were verified by PCR-based genotyping using gene- and locus-specific primer pairs (Supplementary Table S1) as previously described [4].

### 2.3. Light Conditions

*T. atroviride* was cultivated in triplicate on PDA plates at 25 °C for up to 7 days. To this end, six millimeter wide agar plugs derived from the actively growing zone at the colony margins were inoculated at the center of fresh PDA plates and incubated under (a) complete darkness, (b) white light–dark cycles (12:12 h; Economic lux chamber, Snijders Labs, 30 W/m^2^ max intensity), or (c) varying light wavelengths. For the latter purpose, light chambers were designed using LED bulbs emitting blue (light wavelength ≈ 459 nm, luminous intensity 12.85 µW/cm^2^), green (light wavelength ≈ 517 nm, luminous intensity 6.22 µW/cm^2^), red (light wavelength ≈ 630 nm, luminous intensity 2.70 µW/cm^2^), or yellow (light wavelength ≈ 590 nm, luminous intensity 2.00 µW/cm^2^) light. LEDs were positioned at 9 cm vertical height over the fungal colonies. Radial growth rate was determined by measuring colony radii and calculating the radial growth rate (cm/d) for each time point. Conidia were quantified with a hemocytometer after being harvested from a seven days old culture grown on PDA.

### 2.4. Mycoparasitic Activity Assay

Plate confrontation assays of *T. atroviride* against *B. cinerea*, *R. solani*, or *F. graminearum* were performed in triplicate on PDA plates. Fungi were inoculated at a distance of 6 cm and plates incubated at 25 °C for a total of seven days under the different light treatments described above.

### 2.5. Quantification of 6-PP

*T. atroviride* was cultivated in triplicate for 48 h (wild type) or 72 h (Δ*tmk3* mutants due to their slower growth) at 25 °C on cellophane-covered PDA plates under the different defined light regimes to determine the amount of secreted 6-PP. After the mycelia-covered membrane was removed, 1 g of agar from below the fungal colony was harvested and the triplicates pooled. Mycelial dry weight was determined from each membrane to assess biomass production. Five milliliters of the extraction solvent (methanol (MeOH):H_2_O 3:1 (*v*/*v*) + 0.1% formic acid) was combined with 1 g of agar and sonicated for 15 min. MeOH was obtained from Merck, Darmstadt, Germany, water purified with ELGA Purelab Ultra-AN-MK2 from Veolia Water, Vienna, Austria, and formic acid (FA) MS-grade was from Sigma-Aldrich, Vienna, Austria. One milliliter of extract was mixed with 0.5 mL of acidified water containing 0.1% FA to obtain an organic-solvent:water ratio of 1:1 (*v*/*v*). Samples were diluted 1:10 (*v*/*v*) with MeOH:H2O 1:1 (*v*/*v*) + 1% FA for liquid chromatography coupled to high-resolution mass spectrometry (LC-HRMS) analysis using a LC-HRMS system consisting of a Vanquish ultra-high-performance liquid chromatography (UHPLC) coupled to a QExactive Orbitrap HF mass spectrometer (Thermo Fisher Scientific, Bremen, Germany). After injection of 2 µL of the sample, chromatography was performed on a reversed phase C18 column XBridge 150 × 2.1 mm i.d., 3.5 µm (Waters, Milford, MA, USA) using H_2_O and MeOH (both acidified with 0.1% FA) as eluents A and B, respectively. By this, a linear-gradient elution with increasing MeOH content was obtained. The content of methanol was increased to 100% within 9 min (3 min hold) after an initial hold time of 1 min at 10% eluent B before the system was re-equilibrated at 10% eluent B for 7 min (total run time 20 min). A constant flow rate of 0.25 mL/min was applied. Mass spectra from *m*/*z* 100 to 1000 were recorded in positive ionization mode (resolving-power setting of 120,000 at *m*/*z* 200). The XCalibur software (Thermo Fisher Scientific, Bremen, Germany) was used for quantification after external standard calibration of 6-PP (purity > 96%; 1, 5, 10, 100, and 500 µg/L; Sigma-Aldrich, Vienna, Austria) (Supplementary Figures S1 and S2). Linear regression resulted in the following calibration curve: y = 2.36 × 10^6^ + 3.75 × 10^6^x and a determination coefficient (R2) of 0.9986. Obtained 6-PP values were normalized to the mycelial dry weight of each triplicate.

### 2.6. Stress Assays

To analyze stress resistance, conidia collected from 7 days old *Trichoderma* cultures grown at 25 °C under different light conditions were exposed to stress agents. A total of 10^6^ conidia were inoculated on PDA plates containing 108 µM congo red or 31 µM calcofluor white to study cell wall stress resistance, 0.5 M NaCl to study osmotic stress resistance, and 2.5 mM H_2_O_2_ for oxidative stress resistance analysis. Cultures were incubated for two days at 25 °C under light–dark conditions in at least three biological replicates. Mycelial stress resistance was analyzed by inoculating mycelia-covered plugs from pre-cultures grown under different light or dark conditions on PDA plates supplemented with respective stress agents.

### 2.7. Microscopic Analysis

An inverted Nikon optiphot-2 microscope or a Nikon SMZ1500 stereomicroscope was used for imaging of fungal hyphae. Images were captured with a digital camera.

### 2.8. Statistical Analysis

Data were subject to one-way analysis of variation (ANOVA). By using least significance difference (LSD) at *p* = 0.05, treatment means were separated [49]. All analyses were performed using the package IBM SPSS Statistics 24.

## 3. Results

### 3.1. Effect of Different Light Regimes on Colony Extension and Asexual Development of T. atroviride

Light is a factor that influences many cellular processes. In *T. atroviride* IMI206040, blue light has been shown to govern asexual reproduction by employing the Tmk3 MAPK pathway [18]. Based on this, we intended to assess the influence of not only blue light but light of different defined wavelengths on radial growth and conidiation of the *T. atroviride* wild-type strains IMI206040 and P1 as well as of Δ*tmk3* mutants derived thereof.

**Figure 1:**
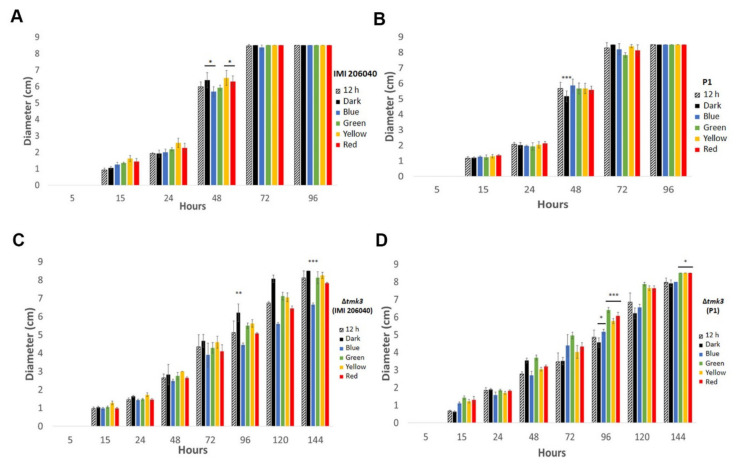
Colony diameters of *Trichoderma atroviride* wild-type strains P1 and IMI206040 as well as their *tmk3*-deficient mutants under different light regimes. Strains IMI206040 (**A**), P1 (**B**), IMI206040-derived Δ*tmk3* (**C**), and P1-derived Δ*tmk3* (**D**) were cultivated on PDA plates in complete darkness, under white light–dark cycles, or in the presence of different light wavelengths. Colony diameters were determined at different time points until the colonies reached the end of the plates (9 cm diameter). The experiment was repeated three times with three replicates each. Asterisks denote significance level: * *p* < 0.05, ** *p* < 0.01, *** *p* < 0.001.

When grown under the different light regimes (complete darkness, white light–dark cycle, or in the presence of either blue, green, yellow, or red light), both wild-type strains developed similar colony diameters irrespective of the applied light conditions tested. Forty-eight hours after inoculation, however, strain IMI206040 had developed slightly larger colonies upon incubation in the dark or when grown in the presence of yellow or red light, while light of shorter wavelengths, such as blue light, negatively impacted colony extension (Figure 1). This effect was not visible with strain P1. Irrespective of the applied light or dark conditions, strain IMI206040 exhibited a slightly enhanced colony extension rate compared to strain P1, reaching the border of the plates after 72 h of growth (Figure 1). Compared to the wild-type strains, the Δ*tmk3* mutant showed a clear growth reduction. Colonies of the IMI206040-derived Δ*tmk3* mutant covered the plates not until 144 h of cultivation in darkness, while light exposure, especially with blue light, led to reduced growth. The P1-derived Δ*tmk3* mutant reached the border of the plates after 144 h only when grown in the presence of green, yellow, or red light, while the colony remained significantly smaller upon growth in complete darkness, white light–dark cycles, and in the presence of blue light (Figure 1).

**Figure 2:**
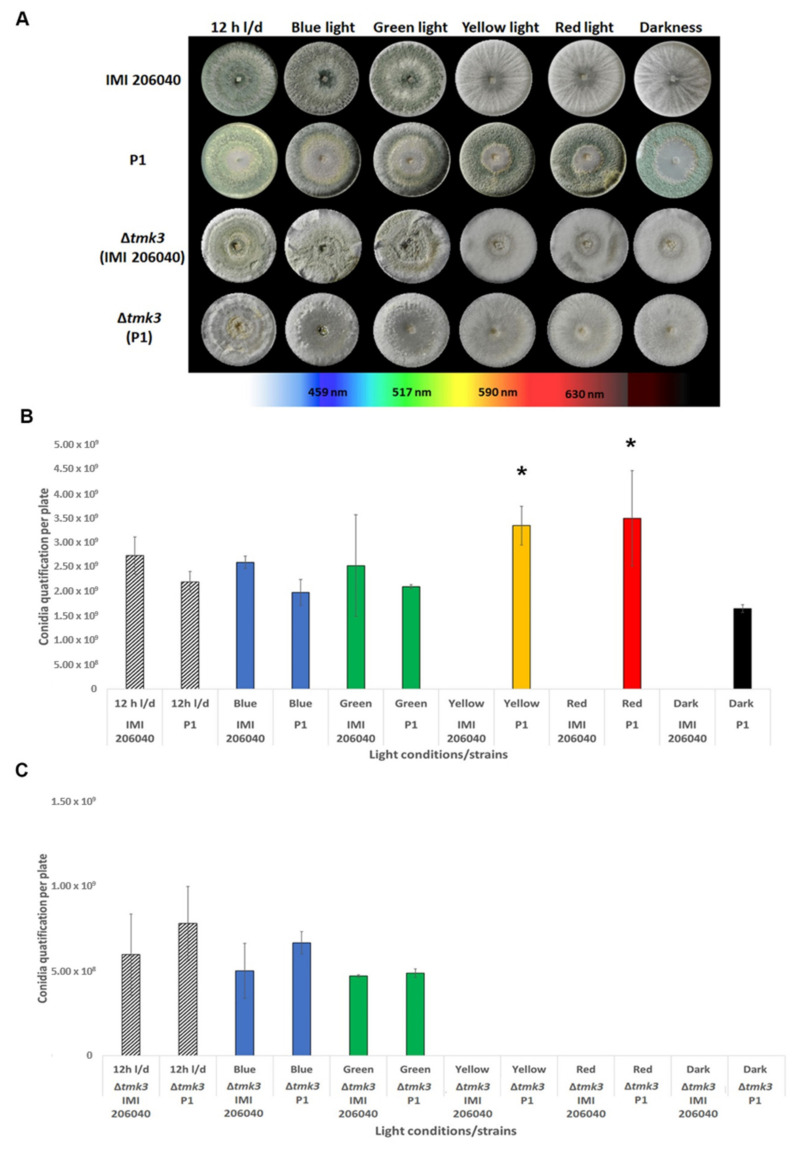
Effect of different types of light exposure on colony phenotypes and conidia production in *T. atroviride* wild-type strains P1 and IMI206040, as well as ∆*tmk3* mutants derived thereof. (**A**) Strain-specific differences in colony morphology upon cultivation for seven days on PDA plates exposed to different light wavelengths, white light–dark cycles (12 h light, 12 h dark), or complete darkness. A representative image of three biological replicates is shown. (**B**,**C**) Quantification of conidia produced by the two *T. atroviride* wild-type strains (**A**) and their ∆*tmk3* mutants (**B**) upon growth under the different light regimes. The experiment was repeated three times with three replicates each. Results shown are means ± SD. *p* values: * *p* < 0.05.

Although colony extension of both *T. atroviride* wild-type strains was only slightly affected by light, massive light-dependent differences in the colony phenotypes became evident between the two strains. *T. atroviride* P1 colonies were green-colored, indicating sporulation, under all conditions tested including growth in complete darkness. In contrast, IMI206040 colonies stayed unpigmented upon exposure to yellow and red light and upon cultivation in darkness, indicating that this strain is only able to conidiate in the presence of white, blue, and green light (Figure 2A). The conidiation behavior of the IMI206040-derived Δ*tmk3* mutant resembled the parental strain, while interestingly deletion of the *tmk3* gene prevented P1-derived mutants to produce green-pigmented conidia in darkness and upon growth in the presence of yellow and red light, a behavior resembling that of the IMI206040 wild-type strain.

To assess whether the observed effects of light on *T. atroviride* conidiation were actually due to alterations in conidia production or only in conidial pigmentation, the number of conidia produced by the different strains under the various light conditions was determined. Upon growth under white light–dark cycles, blue, and green light, *T. atroviride* IMI206040 produced similar numbers of conidia as strain P1, while no conidia could be obtained from IMI206040 colonies grown in the presence of red and yellow light, and in complete darkness. *T. atroviride* strain P1 in contrast produced similar numbers of conidia upon growth in darkness, exposure to blue and green light, and under white light–dark cycles. Interestingly, increased spore densities were observed under yellow and red light in *T. atroviride* P1, suggesting that these light wavelengths additionally trigger conidia production in this strain (Figure 2B). Conidia numbers in the IMI206040- and P1-derived Δ*tmk3* mutants were similar to each other upon growth under white light–dark cycles and in the presence of blue or green light. However, the numbers of conidia from both mutants were massively reduced compared to their respective wild types. In addition, cultures of both Δ*tmk3* mutants were devoid of conidia upon cultivation in darkness and in the presence of yellow or red light, which is similar to the phenotype of the IMI206040 wild-type strain but contrasts the behavior of strain P1.

### 3.2. Assessment of Light-Induced Stress Resistance

**Figure 3:**
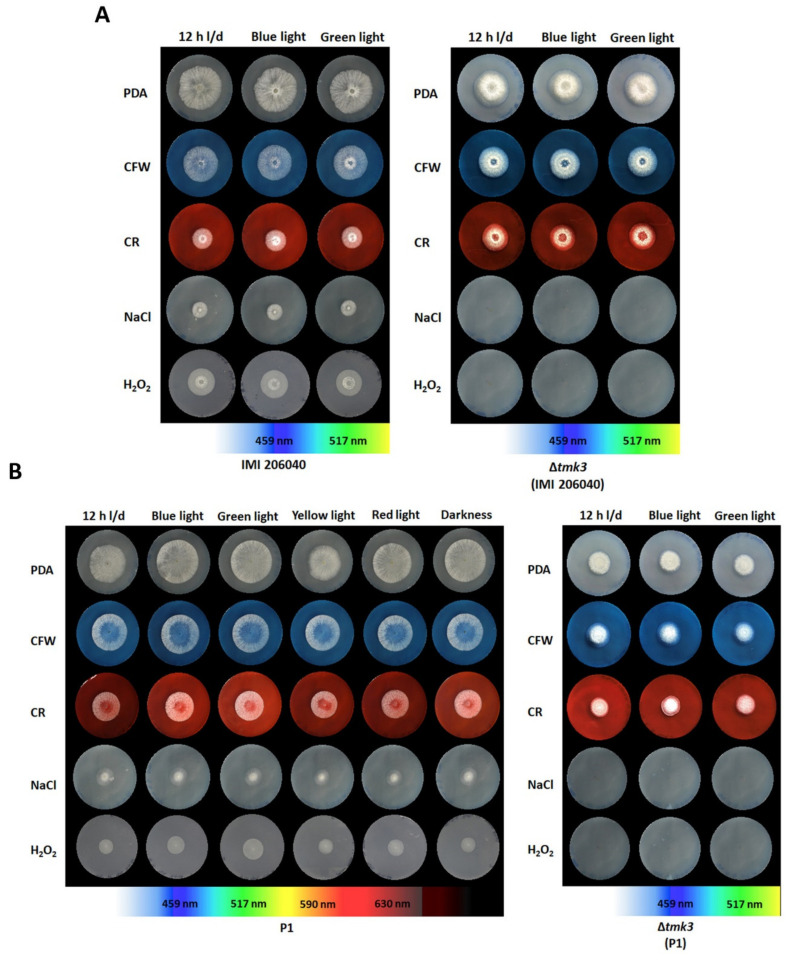
Stress resistance of *T. atroviride* wild-type and Δ*tmk3* mutant colonies developing from conidia previously exposed to different light wavelengths. Colonies of *T. atroviride* IM206040 wild-type and its Δ*tmk3* mutant (**A**) as well as P1 wild-type and its Δ*tmk3* mutant (**B**) developing from conidia gained after seven days of growth in the presence of different light wavelengths, on PDA only (control), PDA with calcofluor white (CFW; 31 µM), PDA with congo red (CR; 108 µM), PDA with sodium chloride (NaCl; 0.5 M), or PDA with hydrogen peroxide (H_2_O_2_; 2.5 mM). Photos were taken after two days of growth at 25 °C under white light–dark cycles (12 h light, 12 h dark).

Photoperception allows fungi to trigger stress resistance in response to light. The exposure of dark-grown *Aspergillus fumigatus* to blue light, for example, resulted in enhanced resistance to UV or hydrogen peroxide mediated stress [10], and growth of *Metarhizium robertsii* in the presence of visible light led to conidia that had higher UV tolerance than that of conidia from dark-grown cultures [50]. As the stress-activated MAP kinase pathway involving Tmk3 has been reported to integrate stress and light signals in *T. atroviride* [18], the effect of different light regimes on cellular stress management in the two *T. atroviride* wild-type and ∆*tmk3* mutant strains was evaluated. All strains tested were able to develop colonies in the presence of 31 µM calcofluor white that were of similar sizes as those grown without stressor (Figure 3). Congo red mediated cell wall stress resulted in moderately reduced colony sizes in both P1 and IMI206040, which however were not affected by loss of *tmk3*. In contrast, both Δ*tmk3* mutants were completely unable to cope with NaCl-mediated osmotic stress, while colony development of the respective wild types was severely inhibited but still possible under this condition. Wild-type strain P1 was more sensitive to oxidative stress triggered by H_2_O_2_ than IMI206040. In addition, both Δ*tmk3* mutants could hardly grow in the presence of this stressor indicated by the fact that development of a colony could only start after 72 h of cultivation. For all strains tested, however, colony development and growth under the tested conditions were completely independent of the previous light exposure. This was even the case with wild-type strain P1 whose conidia derived from light-exposed cultures did not show enhanced resistance to any of the stressors tested compared to conidia produced by dark-grown colonies.

### 3.3. Effect of Different Light Regimes on the Mycoparasitic Activity of T. atroviride Wild Types and ∆tmk3 Mutants

**Figure 4:**
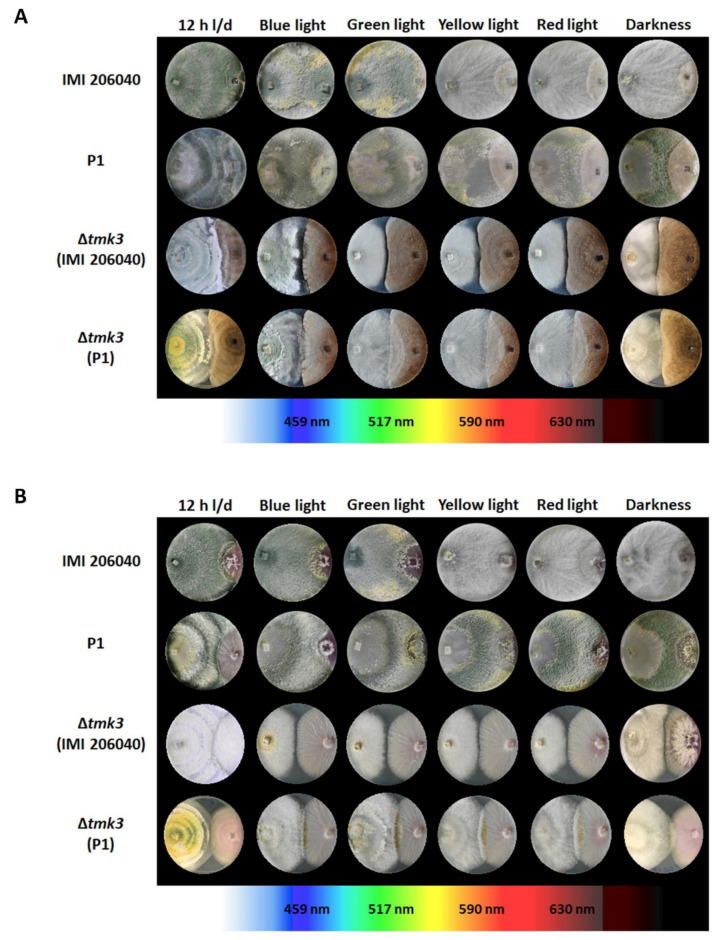
Dual confrontation assays of the *T. atroviride* wild-type strains IMI206040 and P1 and the ∆*tmk3* mutants derived thereof. Co-cultivations with (**A**) *Rhizoctonia solani* (Rs) or (**B**) *Fusarium oxysporum* (Fo) on PDA were performed for seven days in the presence of different light wavelengths, white light–dark cycles (12 h light, 12 h dark), or in complete darkness.

For the analysis of the effect of the different light treatments on the mycoparasitic activity of *T. atroviride,* the two wild types and their ∆*tmk3* mutants were co-cultivated with the host fungi *R. solani* and *F. oxysporum* in plate confrontation assays. Similar to axenic cultures, wild-type strain P1 conidiated in co-cultures with the two tested fungal hosts irrespective of the applied light regime, while conidiation of IMI206040 in the presence of the fungal hosts remained dependent on white, blue, and green light (Figure 4). The ability to antagonize and overgrow *R. solani* only slightly differed between the two *T. atroviride* wild-type strains and emerged to be only marginally affected by the applied light regime. In all cases, both IMI206040 and P1 could completely overgrow the host fungus within seven days. In the interaction with *F. oxysporum*, the mycoparasitic overgrowth ability of both *T. atroviride* wild-type strains was better in darkness and upon yellow or red light exposure, while white light–dark cycle conditions hampered the mycoparasitic attack. Compared to the wild-type strains, both ∆*tmk3* mutants showed a reduced ability to overgrow the fungal hosts. The P1-derived ∆*tmk3* mutant showed partial overgrowth of both hosts under all light conditions tested, while the IMI206040-derived mutant already stopped its growth at the interaction border. This behavior was most evident under blue, green, yellow, and red light conditions where the mutant colonies remained smaller than upon cultivation in darkness or under white light–dark cycle conditions (Figure 4).

### 3.4. Effect of Different Light Regimes on 6-PP Production

**Figure 5:**
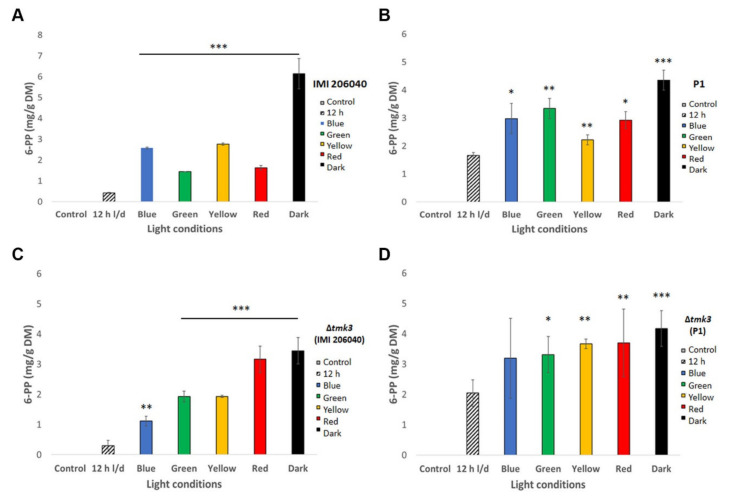
6-PP levels produced by *T. atroviride* wild-type strains IMI206040 and P1, and their ∆*tmk3* mutants upon growth under different light regimes. Amount of secreted 6-PP extracted from the agar of *T. atroviride* wild-type (**A**,**B**) and Δ*tmk3* mutant cultures (**C**,**D**) after growth on PDA in the presence of different light wavelengths, in complete darkness, or white light–dark cycles. Wild-type strains were cultivated for 48 h, while ∆*tmk3* mutants had to be grown for 72 h due to their slower growth rate. The bars represent values normalized to mycelial dry weight (DW). Results shown are means ± SD. *p* values: * *p* < 0.05, ** *p* < 0.01, *** *p* < 0.001.

Both wild-type strains and their ∆*tmk3* mutants were grown on PDA plates under different light conditions in order to evaluate the influence of light on 6-PP production. Secreted 6-PP levels were highest upon cultivation in complete darkness in all strains tested and lowest upon growth in the presence of white light–dark cycles (Figure 5). This inhibitory effect of white light was most evident in wild-type strain IMI206040, which was able to secrete ~6.5 mg 6-PP per gram mycelial dry weight in darkness, but this was below 0.5 mg 6-PP per gram mycelial dry weight upon growth under white light–dark cycles (Figure 5A). Blue, green, yellow, and even red light inhibited 6-PP biosynthesis in both wild-type strains compared to growth in darkness, although to a lower extent than white light cycles. The negative impact of light from across the whole spectrum on the amount of secreted 6-PP was less evident in the Δ*tmk3* mutants than in their respective wild types. Notably, the P1-derived mutant produced similar levels of 6-PP upon growth under blue, green, yellow, and red light than in darkness, and white light–dark cycles only had a minor negative effect on 6-PP biosynthesis in this mutant (Figure 5). In the IMI-derived ∆*tmk3* mutant, the repressive effect of white light treatment was similar than in its wild type.

### 3.5. Comparative Sequence Analyses of the Main Photosensory Proteins of T. atroviride IMI206040 and P1

To assess if the observed differences in the light responses of the wild-type strains IMI206040 and P1 were due to differences in their major photosensory proteins, the protein sequences retrieved from the respective genome databases were compared. Alignment of Blr-1 and Blr-2, phytochrome, and opsin protein sequences revealed a 100% sequence identity between the respective proteins encoded in the two *T. atroviride* strains (Supplementary Figures S3–S5). Hence, the same light sensory proteins seem to govern distinct, strain-specific responses.

## 4. Discussion

In this study, we have shown that light exposure affects the phenotype and behavior of *T. atroviride* in many ways. We found significant differences in the response to light between the two tested *T. atroviride* wild-type strains IMI206040 and P1, and we evaluated the effect of loss of the Tmk3 MAP kinase. Reduction of growth by continuous white light and enhanced radial colony growth in darkness has been previously described for *T. atroviride* IMI206040 [51]. In our experiments, however, cultivation in the presence of white light–dark cycles and light of different wavelengths only had a minor effect on colony extension, but it clearly impacted differentiation. The latter is in accordance with previous reports showing that asexual reproduction of *T. atroviride* IMI206040 is tightly regulated by light [22,45,51,52,53,54]. Accordingly, we found that IMI206040 required light, either white, blue, or green, to trigger conidiation, and the strain produced similar numbers of conidia under these light conditions. In contrast, conidiation of strain P1 occurred in a light-independent way and also in complete darkness. Interestingly, however, yellow and red light seemed to additionally trigger conidia formation in strain P1 as the fungus produced enhanced numbers of conidia compared to the other light conditions tested upon illumination with these wavelengths. Taken together, asexual development is differently affected by light in the two *T. atroviride* strains IMI206040 and P1, suggesting differences in light sensing and/or activation of conidiation-related light-responsive genes. Interestingly, however, comparative sequence analyses of Blr1, Blr2, phytochrome, and opsin proteins encoded in both *T. atroviride* strains revealed a 100% conservation. This indicates that the observed strain-specific responses are not due to differences in these major photosensory proteins but most probably are derived from additional components affecting the response of *T. atroviride* to light. Further studies addressing this issue are needed.

Besides a general growth reduction, deletion of the gene encoding Tmk3 MAP kinase led to significantly less conidia upon growth under white light–dark cycles, blue and green light in both *T. atroviride* strains. These findings are in accordance with a previous study on strain IMI206040 showing that Tmk3 regulates photoconidiation in *T. atroviride* [18]. Interestingly, however, strain P1 lost the ability to conidiate in the dark or in the presence of yellow and red light upon *tmk3* gene deletion, while conidiation in response to white, blue, and green light was still possible in both P1- as well as IMI206040-derived Δ*tmk3* mutants. In IMI206040, both blue light regulators, Blr1 and Blr2, have been found to be required for photoconidiation and the control of light-responsive genes. In addition, Tmk3 has been demonstrated to corroborate with the photoreceptor complex comprising Blr1 and Blr2 in induction of gene expression [18]. *Tmk3* gene transcription as well as phosphorylation of Tmk3 are triggered upon light exposure, and light regulates asexual reproduction through the Tmk3 pathway implying that this MAP kinase is a key player in the light sensing pathway [18]. The role of Tmk3 has hitherto only been studied in strain IMI206040 in response to white and blue light. Our results additionally suggest a connection between Tmk3 and red light signaling, while, interestingly, *tmk3* gene deletion still allowed conidiation in response to blue light.

Similar to other fungi, the high-osmolarity glycerol (HOG) pathway and its Tmk3 MAPK have previously been demonstrated in *T. reesei* and *T. atroviride* IMI206040 to participate in high osmolarity and oxidative stress resistance as well as cell wall integrity [18,39]. In our study, we found a similar role of Tmk3 in *T. atroviride* P1. Respective *tmk3* gene deletion mutants were unable to cope with NaCl-mediated osmotic stress and could hardly develop colonies upon treatment with the oxidative stress-triggering agent H_2_O_2_. However, in the Δ*tmk3* mutants as well as their respective wild-type strains, previous light exposure or absence of light had no significant impact on the resistance to the stressors tested including NaCl (osmotic stress), H_2_O_2_ (oxidative stress), as well as congo red and calcofluor white (cell wall stress). These results somehow contrast previous findings with other fungi, suggesting that visible light acts as a signal for stress [55]. In *A. fumigatus*, for example, the response to visible light included enhanced resistance to UV and oxidative stress and an increased sensitivity to perturbation of the cell wall [10]. Secondary metabolite production is as well among the various processes impacted by light in fungi [56]. The impact of light, however, seems to depend on the fungal species as well as the specific secondary metabolite. While in *Aspergillus flavus*, for example, aflatoxin biosynthesis was negatively affected by light, production of ochratoxin in *Aspergillus ochraceus* was enhanced [57,58]. Biosynthesis of the mycotoxin citrinin by *Penicillium verrucosum* was increased by blue light [59], and toxin production in *Alternaria alternata* was reduced upon blue light irradiation [60,61]. In *Aspergillus niger*, fumonisin production was increased under blue and red light, while ochratoxin levels were reduced compared to dark incubation [62]. In addition, the wavelength of light also impacts fungal secondary metabolite production. In *A. nidulans*, the red light receptor FphA has been reported to suppress mycotoxin biosynthesis, whereas the blue light sensors LreA and LreB had a stimulatory effect [20]. Our results on *T. atroviride* revealed the highest 6-PP levels upon growth under dark conditions in both strains tested, while light, with only a minor influence of the wavelength, negatively affected 6-PP production. In *A. nidulans* and other filamentous ascomycetes, the velvet protein complex comprising VeA, VelB, and LaeA acts as a light-dependent key regulator of development and secondary metabolism. VeA is mainly cytoplasmic in the presence of light, while it is imported into the nucleus in darkness, where the fully functional velvet complex acts as an activator of secondary metabolism-related genes [21]. Our findings that 6-PP production by *T. atroviride* mainly occurs in the dark and is repressed by light suggests regulation by LaeA/Lae1, the global regulator of fungal secondary metabolism [63], and the velvet complex.

In *Trichoderma virens*, another potent mycoparasitic *Trichoderma* species (which, however, is unable to produce 6-PP), the velvet protein Vel1 has been revealed as a key regulator of biocontrol. *Vel1* mutants were impaired in secondary metabolism, mycoparasitism, as well as in their biocontrol efficiency [64]. In our study, mycoparasitic overgrowth of *F. oxysporum* by both *T. atroviride* wild-type strains, IMI206040 and P1, was enhanced under dark conditions as well as in the presence of yellow and red light compared to white light–dark cycles and blue light illumination. In contrast, the mycoparasitic overgrowth of *R. solani* was largely light-independent. 6-PP has previously been shown to exhibit antifungal activity [2], and studies with *T. atroviride* indicated that 6-PP production can be elicited by fungal host secreted metabolites, as observed with *R. solani* [65]. Our results from the plate confrontation assays, however, suggest that 6-PP only plays a minor role in the mycoparasitic interaction. Beneficial effects on plants have been reported for 6-PP, such as enhanced levels of monoethanolamine (MEA) and lycopene in tomato, which results in an optimization of the photosynthesis process and improvement of antioxidant activity [66]. On the other hand, the difficulties of *T. atroviride* to overgrow *F. oxysporum* in the presence of white, blue, and green light might also be due to enhanced production of secondary metabolites by the host fungus under these conditions that inhibit *Trichoderma* growth or protect *F. oxysporum* against *T. atroviride* attack. Accordingly, it has been shown that light increases fumonisin biosynthesis in *Fusarium* spp. [67] and that blue light triggers red pigment content of *F. oxysporum* [68].

In conclusion, this study has shown that *T. atroviride* is able to sense and respond to different light regimes that impact differentiation, mycoparasitic activity, and 6-pentyl-α-pyrone production. In addition, the two *T. atroviride* strains tested, IMI206040 and P1, differed in their light responses, which, however, was not due to differences in their major photosensory proteins such as the Blr1/2 complex, phytochrome, or opsin.

## Supplementary Materials

The following are available online at https://www.mdpi.com/2076-0817/9/10/860/s1, Table S1: Primers used for generation of tmk3 gene deletion cassette and genotyping, Figure S1: Chromatograms of the lowest measured values in samples in comparison with the control and with the lowest measured 6-PP standard concentration, Figure S2: Chromatogram-overlay to show the lowest measured 6-PP standard concentration in comparison with the sample showing the lowest 6-PP concentration, Figure S3: Percentage of sequence identity of fungal light sensors, Figure S4: Sequence alignment of fungal phytochromes and White Collar-like proteins, Figure S5: Sequence alignment of fungal opsins.
